# Molecular Insights into Dynamic Relaxation in Vitrimers: Transesterification-Driven Self-Healing at the Atomistic Level

**DOI:** 10.3390/ma19173702

**Published:** 2026-08-31

**Authors:** Hao Yuan, Austin Knight, Long Jiang, Liangliang Huang

**Affiliations:** 1 School of Sustainable Chemical, Biological and Materials Engineering, University of Oklahoma, Norman, OK 73019, USA; hao.yuan-1@ou.edu; 2 Department of Mechanical Engineering, North Dakota State University, Fargo, ND 58108, USA; austin.knight@ndsu.edu (A.K.); long.jiang@ndsu.edu (L.J.)

**Keywords:** vitrimer, covalent adaptable network, self-healing polymers, bond–exchange reactions, molecular dynamics simulation

## Abstract

Vitrimers are covalent adaptable networks that combine the structural stability of thermosets with self-healing enabled by dynamic covalent bonds. However, the molecular-level interplay among polymerization, prescribed bond-exchange reactions (BERs), finite-time mechanical relaxation, and nanoscale interfacial recovery remains incompletely understood. Here, we employ all-atom molecular dynamics simulations with the REACTER template-based reaction algorithm to construct a Bis-GMA/2-HEMA methacrylate polymer assembly and to model temperature-dependent transesterification. The resulting structure exhibits heterogeneous connectivity and a simulated glass transition temperature consistent with reported trends. With the imposed BER-acceptance window centered near the simulated T_g_, mechanical and self-healing analyses reveal a model-conditioned temperature crossover. Near the glass transition region, BERs cooperate with emerging segmental mobility to accelerate finite-time mechanical relaxation and early-stage healing. At higher temperatures, thermally activated chain mobility and physical interfacial reconsolidation dominate the apparent response, making the incremental contribution of BERs secondary. Because the glass transition temperature and the topology-freezing temperature are distinct and the latter is not calculated here, the near-transition crossover is interpreted as conditional on the adopted BER schedule rather than as a universal vitrimer rule. These results clarify how dynamic covalent chemistry and temperature-controlled relaxation jointly regulate nanoscale vitrimer mechanics and repair within the accessible simulation window.

## 1. Introduction

The thermoset resin is widely applied due to its unique properties such as low shrinkage, low viscosity, long pot life, satisfactory wetting, excellent adhesion to various substrates, and desired thermal and mechanical properties after curing [1,2]. However, the intrinsically irreversible cross-linked structures of conventional thermosets impose fundamental limitations on their recyclability, repairability, and sustainability [3,4]. This limitation creates significant challenges for environmental sustainability, waste management, and long-term economic viability [5,6].

To address these challenges, significant efforts have been devoted to developing recyclable and self-healing thermosets by incorporating reversible bonds into polymer networks. These include both reversible non-covalent interactions and reversible covalent bonds. Thermosets relying on non-covalent interactions are often mechanically fragile and fail under high stress [7]. In contrast, covalent adaptable networks (CANs), or “vitrimers,” first introduced by Leibler and co-workers [8], stand out from traditional polymer systems by combining the structural stability of thermosets with the processability of thermoplastics. While conventional thermosets form permanent cross-linked networks that cannot be reshaped or repaired once cured, vitrimers incorporate dynamic covalent bonds that enable network rearrangement under external stimuli [9,10,11]. This topological adaptability endows vitrimers with self-healing capabilities and recyclability, making them especially appealing for demanding applications [12]. For example, Han et al. [13] pioneered a catalyst-free epoxy vitrimer system based on a hyperbranched epoxy (HBE) and succinic anhydride. The abundant hydroxyl groups in the HBE structure serve both as reaction sites and intrinsic catalysts, enabling dynamic transesterification without the need for metal additives. The resulting vitrimer exhibits rapid stress relaxation, excellent self-healing and weldability, along with glass transition temperatures up to 96 °C and high thermal stability, comparable to traditional epoxy resins. This system also demonstrates outstanding coating performance, including corrosion protection after self-repair, highlighting its potential for structural coating and adhesives in harsh environments. Recently, Ye et al. [14] developed a vitrimer by incorporating a phosphorus- and tertiary amine-containing oligomer into epoxy resin, achieving exceptional mechanical strength and toughness without the use of external catalysts. Importantly, the system supports closed-loop chemical recycling of both the vitrimer matrix and carbon fibers, making it ideal for sustainable composite manufacturing. Despite these advances, the molecular origins of vitrimer behavior, including the interplay among segmental mobility, transesterification kinetics, and network topology, remain difficult to probe experimentally. This is particularly challenging near the glass transition temperature (T_g_), where the rheological response changes abruptly.

Molecular dynamics simulations have therefore become an essential tool for studying vitrimers at the molecular scale [15]. Zhao et al. [16] employed coarse-grained MD simulations to investigate the effects of dynamic bond-exchange reaction (BER) frequency and crosslinking density on stress relaxation, mechanical properties, and the self-healing ability of vitrimers. Their study revealed a delicate balance between network rigidity and topological adaptability, enabling reliable predictions of tensile behavior and shape recovery through multiple reprocessing cycles. In parallel, Chen et al. [17] developed a reactive MD simulation framework coupled with a size extrapolation strategy to quantitatively capture the polymerization kinetics and network evolution of epoxy vitrimer systems. By systematically varying monomer ratios and polymerization temperatures, they elucidated the impact of processing parameters on crosslink density and dynamic BER efficiency. These simulation-based investigations provide critical molecular-scale insights for optimizing vitrimer design, linking reaction mechanisms to macroscopic properties such as recyclability and mechanical robustness. However, most MD studies treat polymerization and bond exchange separately and mainly examine how temperature influences vitrimer mechanical or healing behavior. A unified, mechanistic understanding of how temperature and BER jointly regulate vitrimer performance at the molecular level remains largely unexplored.

In this study, we develop a fully atomistic molecular dynamics framework for a methacrylate vitrimer model based on bisphenol A-glycidyl methacrylate (Bis-GMA) and 2-hydroxyethyl methacrylate (2-HEMA). The model explicitly captures primary polymerization followed by template-based transesterification using REACTER. Rather than focusing solely on equilibrium mechanical properties, we investigate how finite-time bond exchange, segmental mobility, and interfacial relaxation collectively govern mechanical deformation and nanoscale self-healing. We explicitly distinguish the calculated T_g_ from the operational topology-freezing temperature T_v_, which is not determined in the present simulations, and define T_BER_ as the reference temperature of the imposed BER-acceptance schedule. By comparing reactive and nonreactive systems across glassy, transition, and high-temperature regimes, we establish a model-conditioned mechanistic picture linking dynamic covalent chemistry with simulation-timescale viscoelastic relaxation and structural recovery.

## 2. Simulation Methodology

Bis-GMA is a widely used methacrylate resin owing to its excellent mechanical strength, good processability, and well-characterized molecular structure, while 2-HEMA, a low-molecular-weight monomer, is employed as a diluent to reduce viscosity and improve flow and wetting behavior [18]. The initial system comprised 300 Bis-GMA monomers and 600 2-HEMA monomers, corresponding to a molar ratio of 1:2. All-atom molecular models of Bis-GMA and 2-HEMA monomers were constructed using the Materials Studio software package [19] based on their established chemical structures, and the initial system configuration was prepared using Packmol [20,21] to ensure homogeneous spatial distribution. All molecular visualizations were produced using OVITO version 3.15.0 [22]. The system was initially equilibrated under the NPT ensemble at 300 K and 1 atm, using a Nose–Hoover thermostat [23] to achieve thermal relaxation. After equilibration, the resulting monomeric mixture served as the precursor for polymerization.

All molecular interactions were described using the OPLS-AA all-atom force field [24,25]. Complete parameters are provided in Table S1. Nonbonded interactions were modeled with a 14 Å Lennard-Jones and Coulomb cutoff, and long-range electrostatics were treated with the particle–particle particle–mesh method [26]. Atomic partial charges were uniformly scaled by 0.8 [27], and the hydroxyl charge modification used for one Bis-GMA atom type is documented in the Supplementary Information. As system-level checks, the equilibrated uncured 1:2 Bis-GMA/2-HEMA mixture had a density of 1.08 g cm^−3^ at 300 K and 1 atm, compared with an ideal-volume estimate of 1.13 g cm^−3^, calculated from reported pure-component densities of 1.16 and 1.07 g cm^−3^, respectively (a deviation of approximately −4.3%) [28]. The simulated T_g_ of 394 K also lies within the reported 383–433 K range for related vinyl-ester thermosets [29]. These checks support semi-quantitative interpretation of packing and thermal trends, but they do not validate absolute reaction barriers, reaction rates, or all hydrogen-bond energetics. Following energy minimization, velocities were initialized from a Maxwell–Boltzmann distribution at 300 K. Simulations were performed using LAMMPS version 2Aug 2023 [30].

Polymerization of the Bis-GMA and 2-HEMA mixture was modeled using the REACTER module in LAMMPS version 2Aug 2023 [31]. Pre- and post-reaction templates with explicit atom mappings were used to update topology when reactive sites met geometric and probabilistic criteria; representative structures are shown in Figure S1. Templates for primary monomer–monomer coupling and secondary coupling between monomers and growing chains described vinyl-group reactions, with a 4.0 Å carbon–carbon cutoff. The REACTER acceptance probability was set to p = 1.0. This parameter determines whether a geometrically and topologically eligible event is accepted; it is not a physical kinetic rate constant. The value p = 1.0 was chosen to obtain a highly reacted configuration within the accessible simulation time and should therefore be interpreted as part of a network-construction protocol rather than a prediction of experimental polymerization kinetics. For a sequence of N eligible trials, the Bernoulli gate gives E[K] = pN accepted events; equivalently, the expected number of eligible trials required for K accepted events is K/p. Thus, in the ideal independent-trial limit, p = 0.50 and 0.25 require approximately two and four times as many eligible trials as p = 1.0 to reach the same accepted-event count. This is an algorithmic sensitivity, not a kinetic rate law, and it does not guarantee matched-conversion topology equivalence because every accepted event changes the geometry and future eligibility of the system (Figure S3 and Table S4).

Under NPT conditions at 300 K and 1 atm, the system was simulated for 5 ns. At the endpoint, approximately 99% of monomer molecules had participated in at least one vinyl-coupling event. This metric does not mean that 99% of all vinyl groups reacted and does not imply formation of a single percolated covalent network. Graph analysis identified 247 connected polymer clusters in the polymerized configuration; accordingly, we refer to the product as a heterogeneous polymerized assembly. Because the calculation begins with 900 monomer components, reduction to 247 connected components requires at least 900 − 247 = 653 accepted inter-component coupling events. Intracomponent cyclization can add accepted events without reducing the component count, so 653 is a rigorous lower bound rather than the total event count. The archived revision files do not contain the intermediate fix bond/react history, and no time-resolved event trajectory is reconstructed from the endpoint data. Supplementary Information Section S4 summarizes the exact endpoint accounting; Figure S3 and Table S4 separately present the analytical acceptance-probability sensitivity. The 5 ns interval is therefore treated as a network-construction interval, not as a prediction of experimental polymerization kinetics. At equal simulation time, a lower p necessarily reduces the expected number of accepted events; at matched reacted-monomer fraction, altered event ordering may still change cluster topology. All structural and property results are therefore explicitly conditioned on the adopted *p* = 1.0 construction.

**Figure 1:**
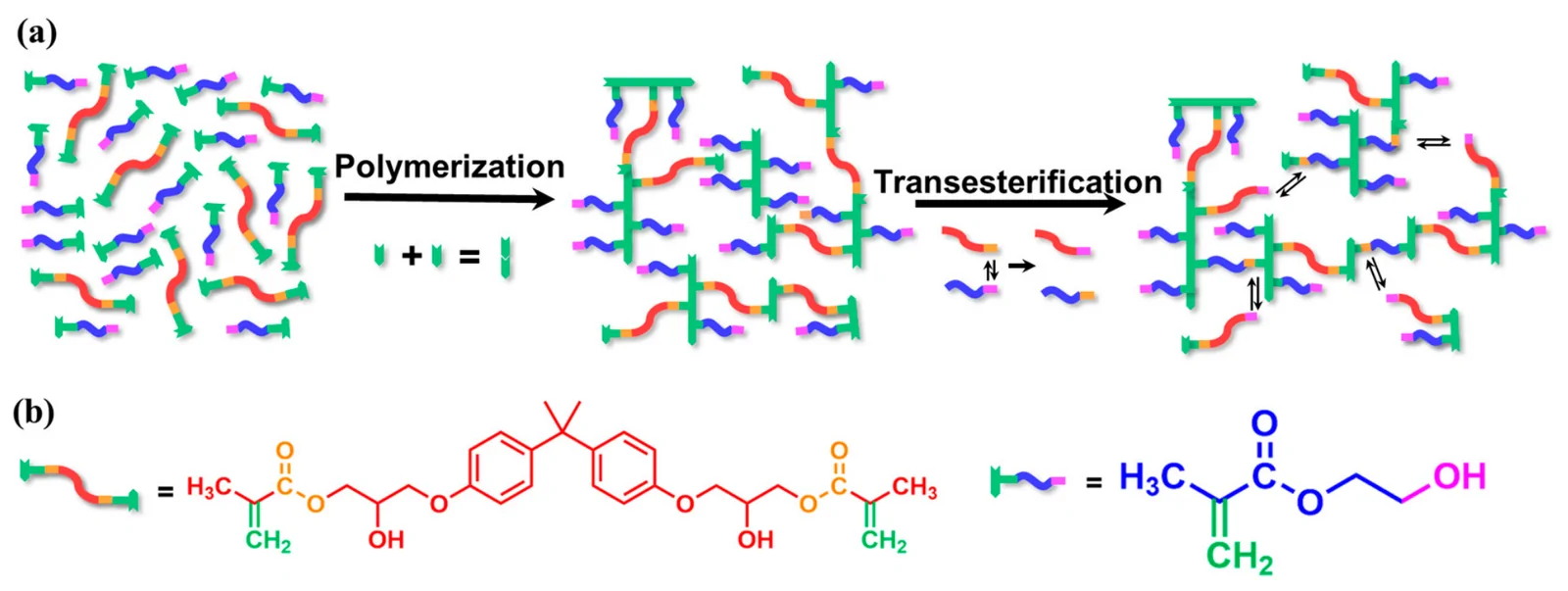
(**a**) Schematic illustration of Bis-GMA/2-HEMA polymerization followed by transesterification. (**b**) Molecular structures of Bis-GMA (red, with ester groups highlighted in yellow) and 2-HEMA (blue, with the hydroxyl group highlighted in orange). Carbon–carbon double bonds that serve as polymerization sites are highlighted in green. In panel (**a**), distinct chain colors are used only to distinguish polymer segments and do not encode chemical identity.

After polymerization, the resulting assembly was used as the starting structure for a separate temperature-dependent transesterification stage. In this sequential protocol, associative exchange does not change the endpoint reacted-monomer fraction or the total number of bonds established during polymerization. Instead, it redistributes connectivity among pre-existing segments, modifying local architecture, connected-component membership, and stress-relaxation pathways. The sequential design isolates post-polymerization BER effects; possible coupling between polymerization and transesterification during curing remains beyond the present scope. The sequential polymerization and transesterification workflow is summarized in Figure 1.

Transesterification responsible for topology rearrangement was implemented with a second set of REACTER templates for associative exchange between ester and hydroxyl functionalities. A 2.82 Å cutoff was used to identify eligible reactive pairs, and the acceptance probability was prescribed as a continuous function of temperature. The glass transition temperature T_g_ and topology-freezing temperature T_v_ are physically and operationally distinct: T_g_ characterizes segmental vitrification, whereas T_v_ is commonly inferred from exchange-controlled viscosity or stress relaxation on a specified observation timescale and may lie above or below T_g_ depending on chemistry and kinetics [32,33,34]. The present work does not determine T_v_ because no long-time viscosity or stress-relaxation extrapolation is performed. We therefore use T_BER_ to denote the reference temperature of the imposed BER-acceptance function. The production schedule used T_BER_ = 400 K, p_TBER_ = 0.90, and w = 20 K. Because the fitted T_g_ is 394 K, T_BER_ lies 6 K above T_g_ and places appreciable BER activity within the same broad transition region. This proximity is a deliberate modeling approximation and should not be interpreted as evidence that T_v_ = T_g_ for the Bis-GMA/2-HEMA chemistry. The acceptance probability is expressed as

(1) $$p(T)=\frac{1}{exp\left(-a\left(T-T_{BER}\right)-ln\left(\frac{p_{T_{BER}}}{1-p_{T_{BER}}}\right)\right)+1}$$

**Figure 2:**
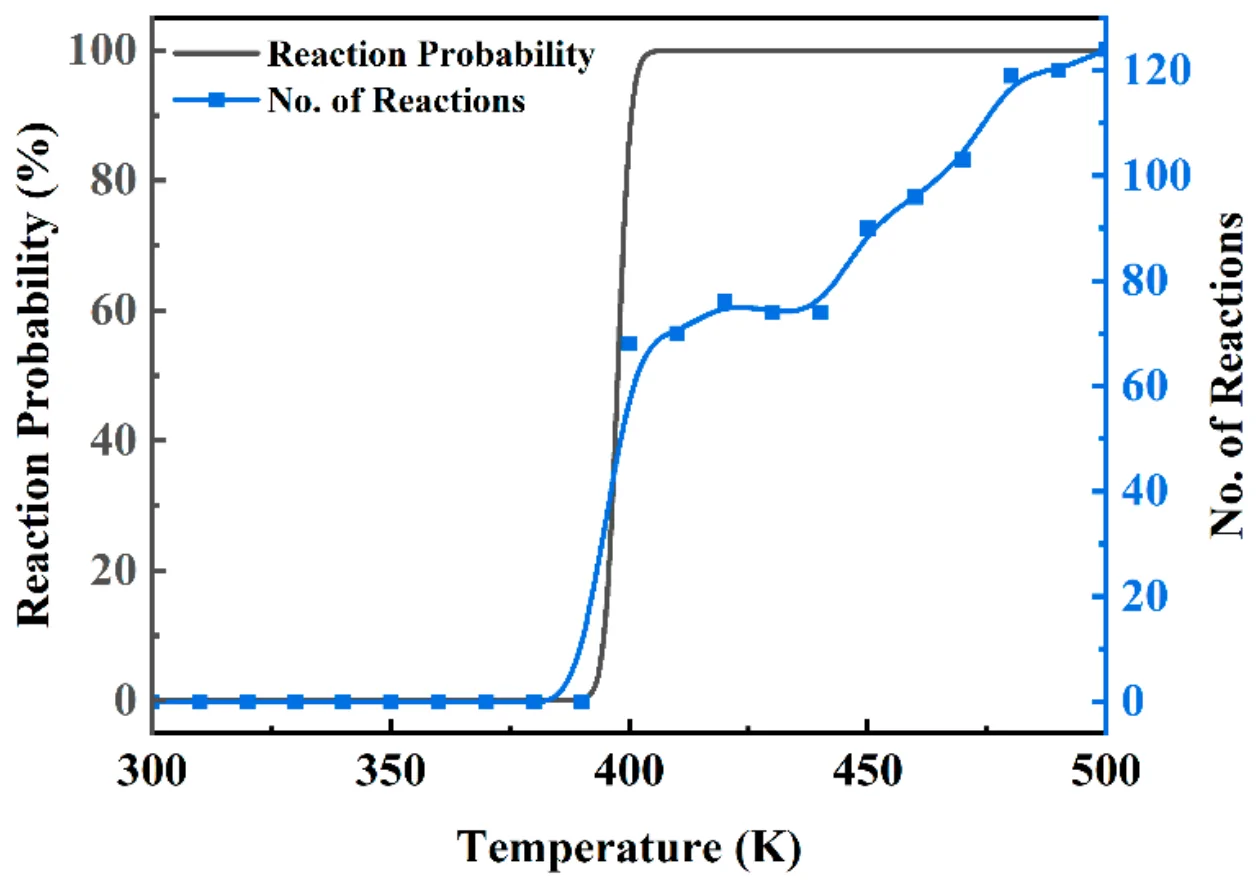
Baseline imposed BER-acceptance probability and observed number of accepted transesterification events within 100 ps as functions of temperature. The probability curve is a model input; the reaction count is the simulation output. The black/gray curve denotes the prescribed acceptance probability on the left axis, whereas blue squares connected by a blue line denote accepted-event counts on the right axis.

Here, p_TBER_ = 0.90 denotes the prescribed acceptance probability at T_BER_ = 400 K. The steepness parameter follows the submitted schedule definition, a = 2p_TBER_/[w(1 − p_TBER_)] = 0.90 K^−1^, and the width parameter w was set to 20 K. Figure 2 reports both the imposed probability and the number of accepted transesterification events observed within 100 ps. The probability is a model input, whereas the reaction count is the simulation output. Because T_BER_ enters Equation (1) as a temperature offset, changing it translates the BER-acceptance curve relative to the independently determined T_g_. Corrected Figure S2 and Table S3 illustrate this input-function sensitivity by shifting the production T_BER_ by −40 and +40 K while retaining p_TBER_ and w. These curves are analytical input translations, not additional mechanical or healing trajectories.

The T_g_ was determined through a stepped cooling protocol. The polymerized vitrimer system was equilibrated at 600 K, followed by gradual cooling to 200 K in 20 K increments. At each temperature, the specific volume was computed after equilibration. The thermal expansion coefficient was determined using

(2) $$a=\frac{1}{V}{\left(\frac{\partial V}{\partial T}\right)}_{P}$$

By fitting linear regimes in the glassy and rubbery states and identifying their intersection, we obtained the simulated value of T_g_.

Uniaxial tensile simulations were performed by stretching the box along x at a constant engineering strain rate of 1 × 10^−5^ fs^−1^ (1 × 10^10^ s^−1^), while the transverse directions relaxed at zero lateral pressure. Young’s modulus was extracted from the initial linear stress–strain regime and is interpreted as an apparent finite-rate tensile modulus rather than an equilibrium rubbery plateau modulus. Tests with and without BER were performed at multiple temperatures to isolate the incremental response associated with the prescribed exchange process on the simulation timescale.

Mechanical damage was introduced by stretching the polymerized assembly beyond fracture. For all healing simulations, the same fractured configuration was used as the initial state; the opposing surfaces were brought into contact under gentle compression and relaxed for up to 5 ns at selected temperatures, following a strategy adapted from previous simulations [35,36,37]. Because the interface spans only a few nanometers, the reported healing efficiency measures recovery of the apparent small-strain modulus across a nanoscale interface and does not imply restoration of the original bulk topology or macroscopic fracture resistance. A second tensile test was used to calculate the healing efficiency:

(3) $$\eta =\frac{E_{healed}}{E_{original}}$$

In addition, the time evolution of van der Waals interaction energy, system density, reaction count, and reaction rate was monitored to characterize interfacial relaxation and network reorganization during self-healing.

All temperature and BER comparisons were made from the same polymerized topology, and all healing comparisons used the same fractured configuration. This constitutes a controlled within-realization paired design: the starting network is held fixed while temperature or BER status is changed. The design isolates condition-dependent differences within one representative assembly and avoids confounding those comparisons with network-realization differences. It does not, however, estimate variability across independently packed and cured networks. Accordingly, no population-level statistical significance is claimed. The whiskers retained in the legacy plots are treated only as descriptive variability markers from the original post-processing and are not interpreted as standard deviations across independent network realizations. The scope of this paired design is stated explicitly here and reiterated in the relevant figure captions.

## 3. Results and Discussion

### 3.1. Structural Properties

**Figure 3:**
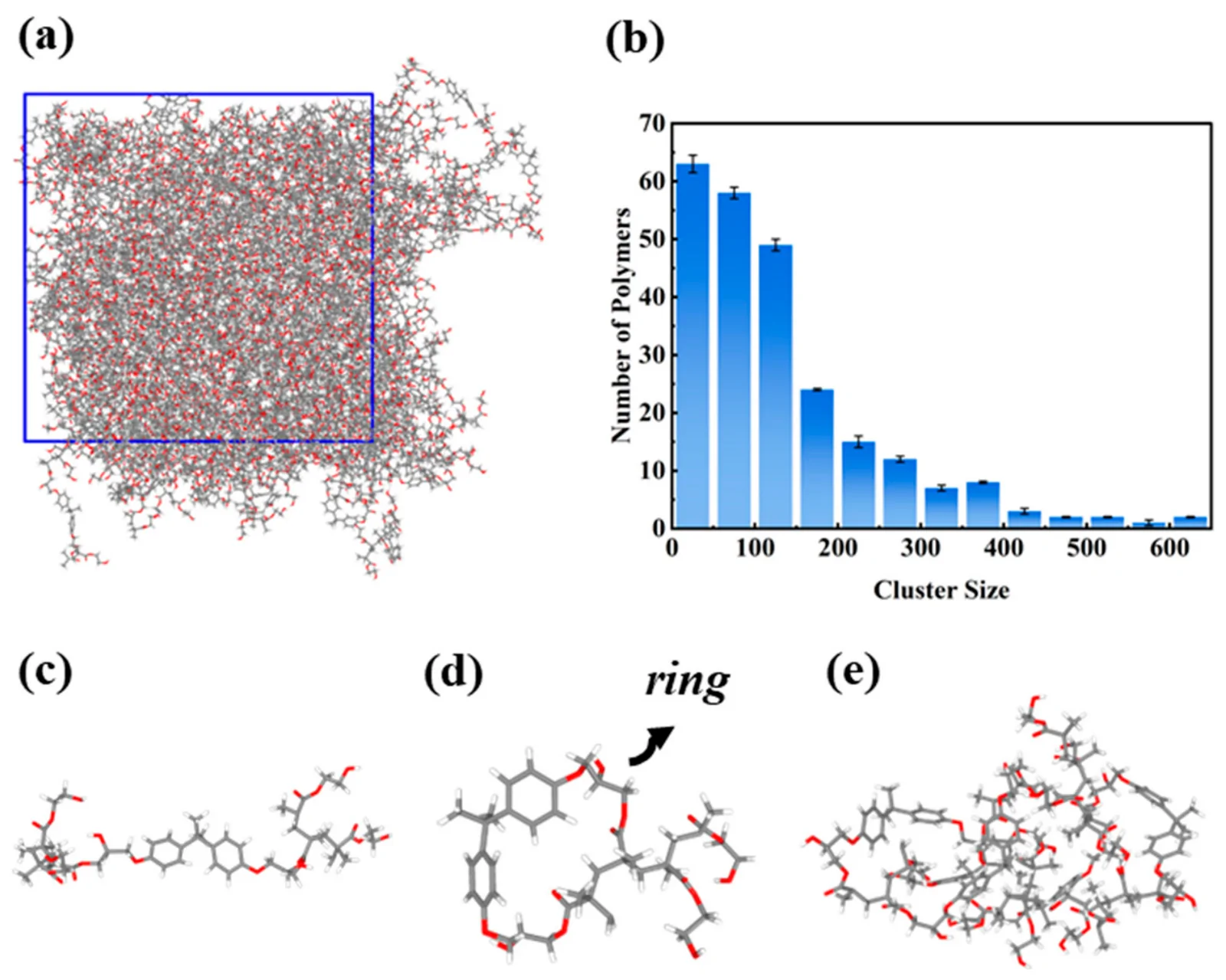
(**a**) Final configuration unwrapped from the 6.9 nm simulation box. (**b**) Connected-cluster size distribution after 5.0 ns of polymerization. Representative topological motifs: (**c**) chain-like, (**d**) cyclic, and (**e**) mixed architectures. Atom colors are carbon gray, oxygen red, and hydrogen white; the periodic simulation box is outlined in blue. In panel (**b**), blue bars show cluster counts and black whiskers are descriptive variability markers from the original post-processing.

The evolution of the polymerized assembly was examined through density and connectivity. Early vinyl-coupling events form dimers and oligomers that subsequently grow through additional reactions. Polymerization increases the density from 1.08 to 1.17 g cm^−3^, consistent with reduced free volume. At 5 ns, approximately 99% of monomer molecules had participated in at least one coupling event, but the product was not a single connected network: graph analysis identified 247 connected polymer clusters. Starting from 900 monomer components, this endpoint requires at least 653 inter-component coupling events, with any intracomponent cyclization increasing the total event count above that lower bound (Supplementary Information Section S4). The final unwrapped configuration and connected-cluster histogram are shown in Figure 3a,b. The broad cluster-size distribution therefore reflects heterogeneous chain growth rather than complete percolation. Only 23 clusters contain more than 300 atoms. Widespread initiation produces many simultaneously growing oligomers and contributes to the broad distribution; fewer initiation sites would be expected to favor longer chains before termination [31].

Beyond chain-length heterogeneity, the polymerized assembly contains chain-like, cyclic, and mixed topological motifs (Figure 3c–e). Their coexistence reflects the stochastic template-based polymerization process. Any eligible carbon–carbon double bond within the reaction cutoff may participate, producing connected components with different sizes and local architectures.

**Figure 4:**
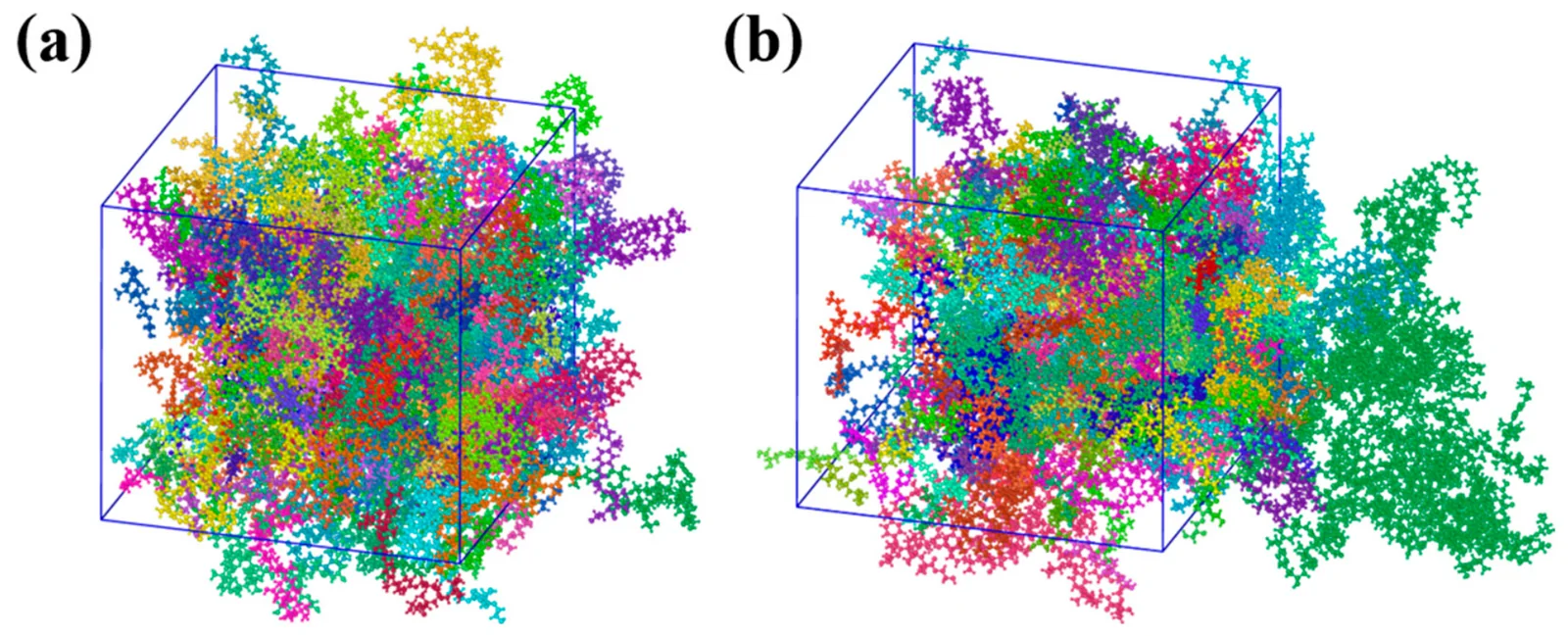
Representative snapshots of the vitrimer network before and after BER-enabled relaxation at 400 K for 5 ns: (**a**) before BERs and (**b**) after BERs. Each connected polymer component is assigned a distinct arbitrary color solely to distinguish connectivity; the colors do not encode chemical identity or a quantitative property. Blue lines outline the periodic simulation box.

Short-cycle formation is usually difficult to observe in atomistic polymer simulations because reptation and loop-closure timescales are long [31]. In the present model, the symmetric Bis-GMA molecule carries two terminal methacrylate groups, which can facilitate intramolecular or local loop closure within the finite simulation window. The observed cyclic motifs should therefore be interpreted within the p = 1.0 construction protocol. The analytical acceptance sensitivity in Figure S3 shows how lowering p lengthens the expected number of eligible trials, but it does not demonstrate that independently generated matched-conversion topologies would be identical. The topology redistribution produced by BER-enabled relaxation is illustrated in Figure 4.

To visualize the structural effect of BER, Figure 4 compares the same polymerized assembly before and after 5 ns of BER-enabled relaxation at 400 K. The number of connected components decreases from 247 to 173. This change does not represent depolymerization or additional vinyl conversion; associative exchange preserves the total bond count while redistributing connectivity, which can merge some components and reorganize local segment architecture. The comparison therefore provides structural evidence of topology reshuffling under the adopted exchange templates.

### 3.2. Glass Transition Temperature

**Figure 5:**
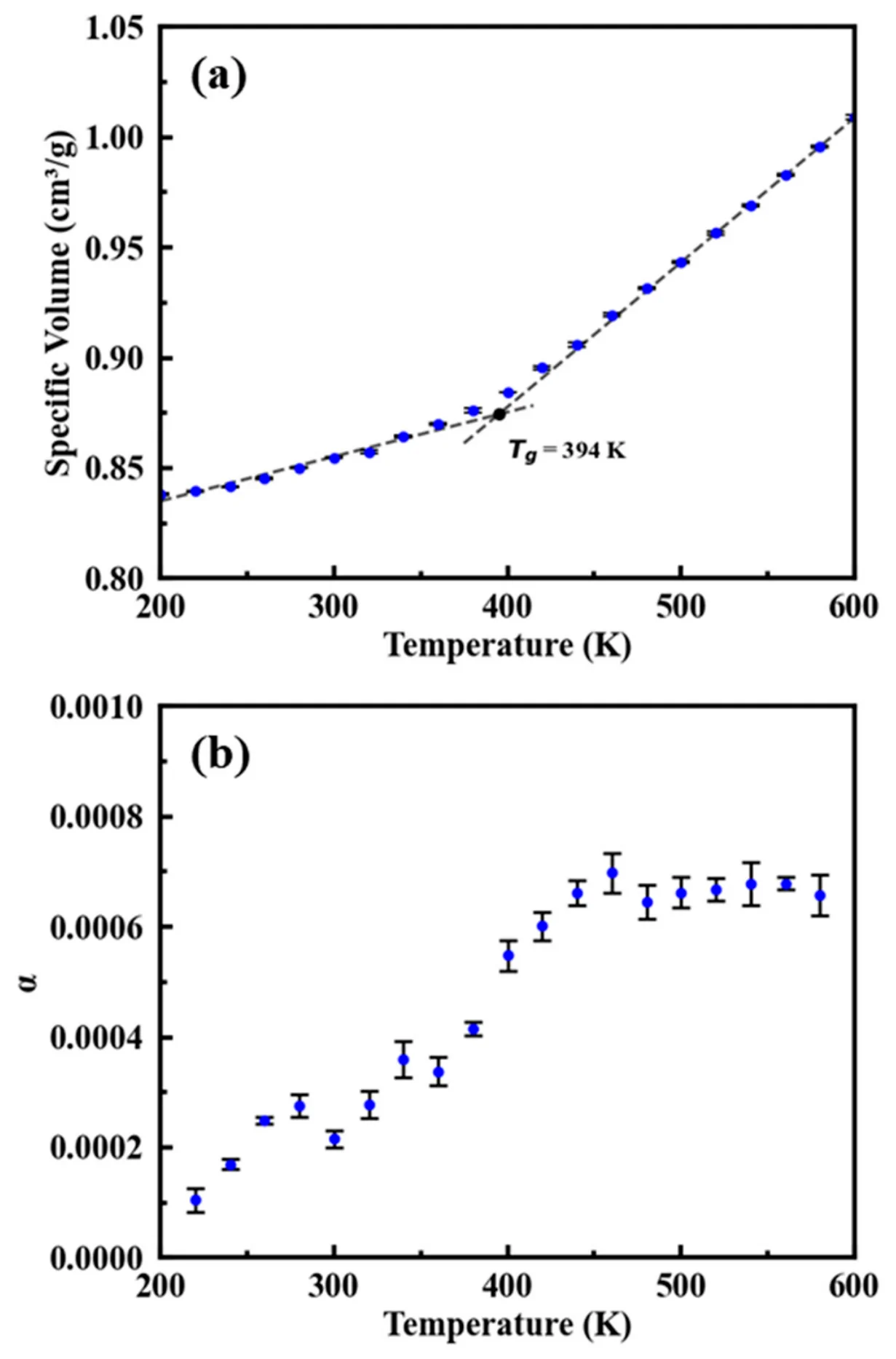
(**a**) Specific volume V and linear fits used to estimate T_g_; (**b**) thermal expansion coefficient α as a function of temperature. Blue symbols are simulation values; black dashed lines in panel (**a**) are the glassy- and high-temperature linear fits, and black whiskers in panel (**b**) are descriptive variability markers from the original post-processing.

The glass transition temperature T_g_ provides a thermal reference for cooperative segmental mobility. The stepwise-cooling specific-volume curve decreases monotonically with temperature (Figure 5a). The thermal expansion coefficient α (Figure 5b) distinguishes a high-temperature mobile regime, a broad transition regime, and a low-temperature glassy regime. This analysis identifies T_g_ only; it does not determine the operational topology-freezing temperature T_v_.

The intersection of the glassy- and high-temperature linear fits gives T_g_ ≈ 394 K (Figure 5a) within the 383 K–433 K range reported for related vinyl-ester thermosets [29]. Agreement with this broad experimental range is a useful system-level check on the force-field packing and thermal response, but it should not be interpreted as validation of the prescribed BER kinetics or as evidence for a particular T_v_.

### 3.3. Mechanical Properties

**Figure 6:**
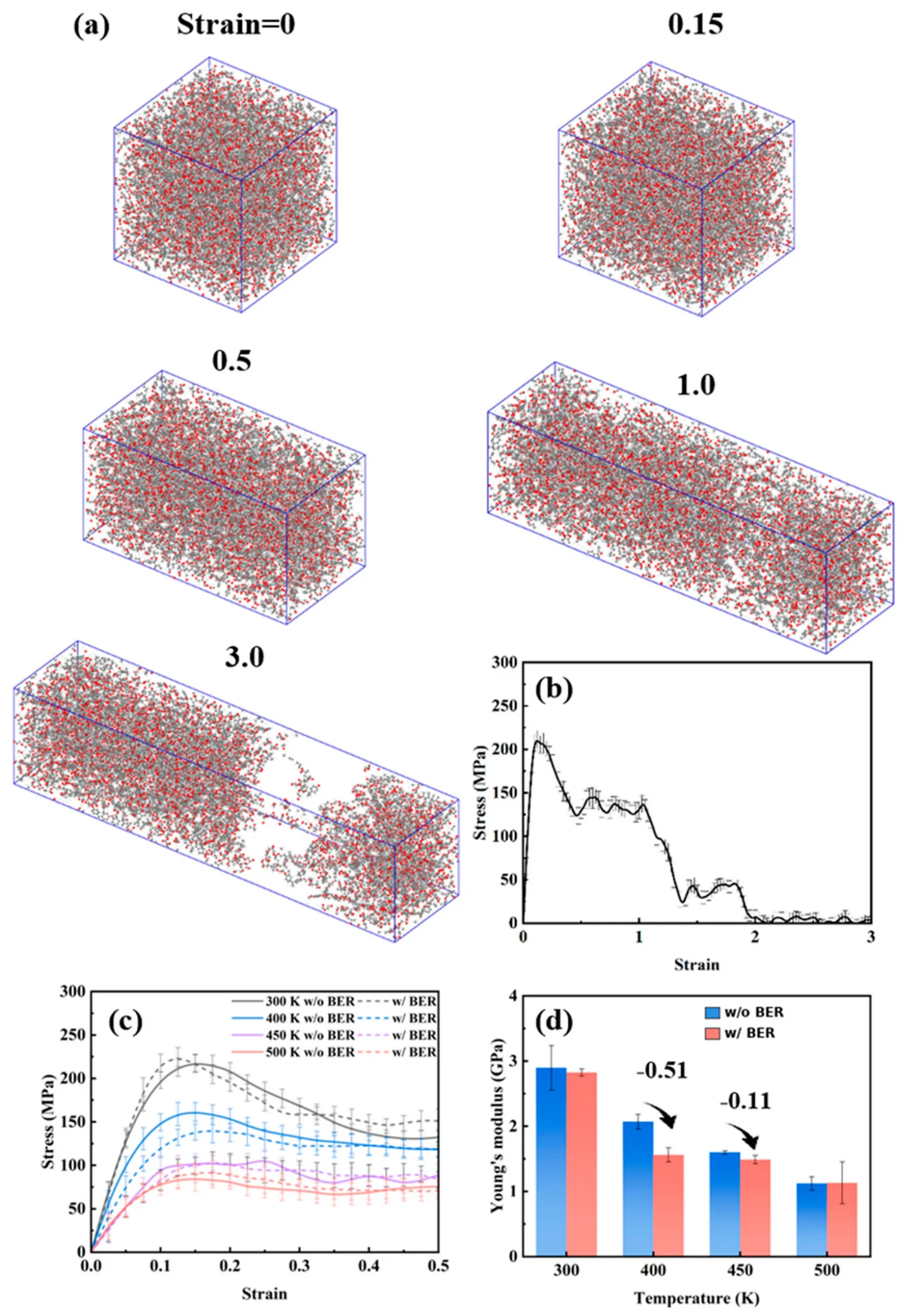
(**a**) Representative structures during uniaxial deformation. (**b**) Stress–strain response at 300 K. (**c**) Stress–strain curves at 300 K, 400 K, 450 K, and 500 K with BER disabled (solid lines) or enabled (dashed lines). (**d**) Apparent Young’s modulus from the initial linear regime; blue bars denote BER disabled and red bars denote BER enabled. All conditions use the same polymerized topology. Whiskers are retained as descriptive variability markers from the original post-processing and are not between-network standard deviations. Atom colors in panel (**a**) are carbon gray, oxygen red, and hydrogen white, with the simulation cell outlined in blue. In panel (**b**), the stress–strain curve is black with gray whiskers. In panel (**c**), 300 K, 400 K, 450 K, and 500 K are black/gray, blue, purple, and red, respectively.

The mechanical response was examined by uniaxial tension with emphasis on temperature and BER. Figure 6a,b relate representative structural states to the 300 K stress–strain curve. The initial linear regime corresponds to an intact assembly under approximately affine deformation; yielding and subsequent stress drops reflect local rearrangement and loss of load-bearing connectivity. At large strain, cavity nucleation and rupture dominate. At elevated temperature, the response becomes more relaxed and flow-like on the MD timescale because segmental relaxation proceeds more rapidly before catastrophic separation.

Figure 6c compares stress–strain curves with and without BER at 300, 400, 450, and 500 K, and Figure 6d compares the corresponding apparent Young’s moduli. Increasing temperature reduces stiffness and peak stress, consistent with accelerated finite-time relaxation. At the applied strain rate, a strain of 0.5 is reached in 50 ps; together with the 100 ps BER counts in Figure 2, this shows that deformation and exchange can occur on comparable simulation timescales near and above the imposed activation window. The high-temperature response is therefore interpreted as finite-time viscoelastic deformation rather than an equilibrated rubbery plateau.

The incremental mechanical effect of BER is temperature-dependent. At 300 K, restricted segmental motion and the near-zero imposed acceptance probability make reactive and nonreactive moduli nearly identical. At 400 K, close to both the simulated T_g_ and the production T_BER_, exchange can cooperate with emerging segmental mobility to promote local topology rearrangement and finite-time stress relaxation, producing a 0.51 GPa reduction in the apparent modulus relative to the nonreactive control. The corresponding reduction is 0.11 GPa at 450 K, and the central values converge at 500 K, where thermal relaxation already dominates. Importantly, this result does not establish a universal rule that BERs are most important at T_g_. It reflects the overlap in the production model between segmental mobility and an imposed BER schedule centered near T_g_.

Importantly, the decrease in modulus with increasing temperature, particularly in the nonreactive control system, should not be interpreted as a contradiction of the entropic elasticity expected for an ideal permanent rubber network. The present simulations probe a high-strain-rate, finite-time tensile response on the atomistic simulation timescale rather than the equilibrium rubbery plateau modulus. Therefore, the observed modulus reduction primarily reflects thermal softening and finite-time viscoelastic relaxation near and above T_g_. Accordingly, the 400 K–500 K results should be regarded as simulation-timescale viscoelastic deformation behavior rather than a fully equilibrated rubbery response.

Changing T_BER_ would alter that overlap without changing the independently calculated T_g_. If T_BER_ were shifted above T_g_, the incremental BER-sensitive window would be expected to move to a higher temperature, and the near-T_g_ response would approach the nonreactive control. If T_BER_ were shifted below T_g_, exchange attempts would become available earlier, but their observable mechanical effect could remain limited until segmental mobility and reactive-site encounters increase. At a sufficiently high temperature, rapid physical relaxation would again reduce the incremental effect of exchange. Figure S2 and Table S3 summarize this model-input sensitivity.

### 3.4. Self-Healing Behavior

**Figure 7:**
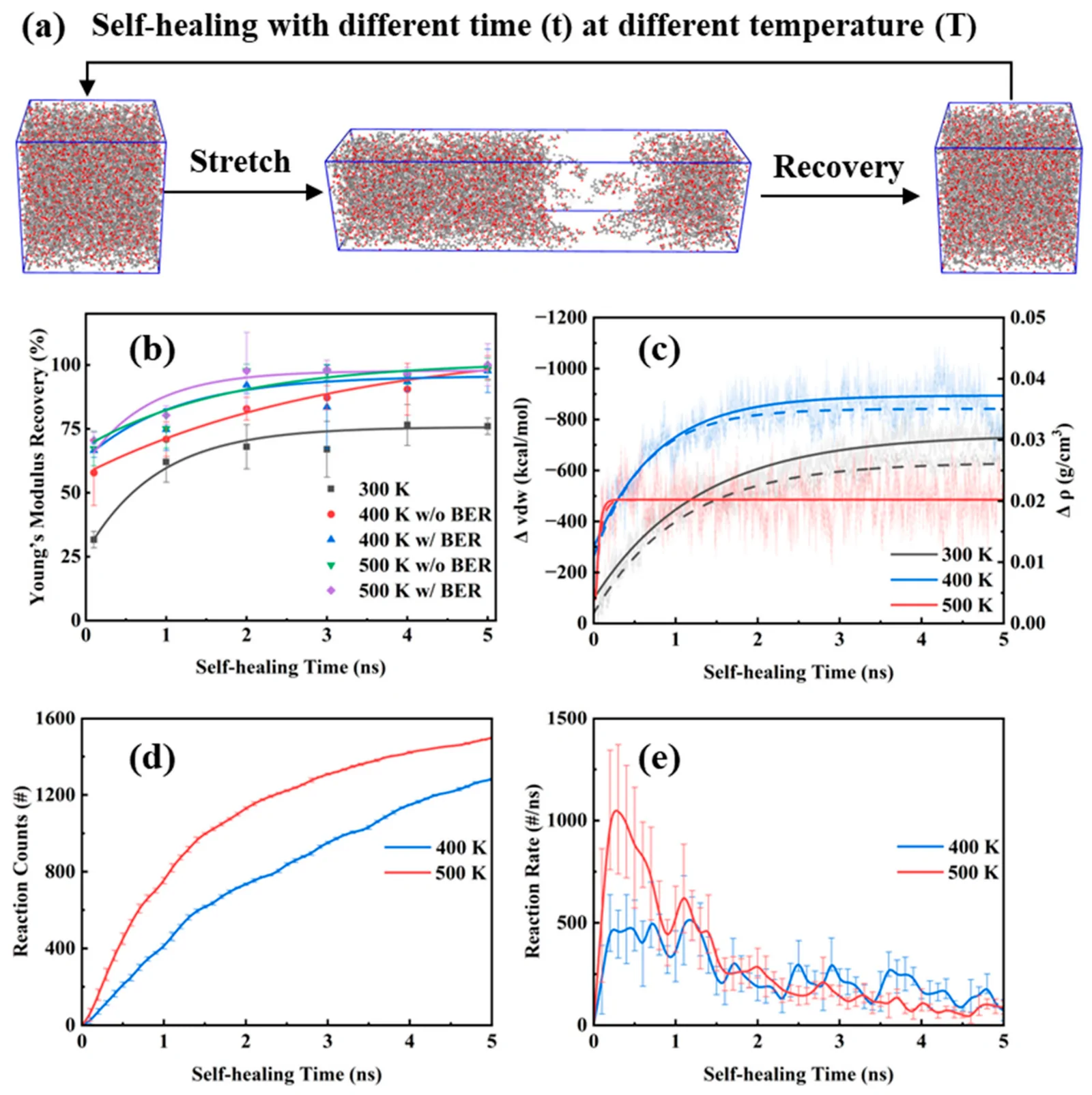
(**a**) Nanoscale self-healing protocol. (**b**) Recovery of apparent Young’s modulus with healing time under the indicated temperatures and BER conditions. (**c**) van der Waals interaction-energy change (solid lines) and density change (dashed lines) during nonreactive relaxation. (**d**) Cumulative accepted BER events and (**e**) BER event rates at 400 and 500 K. All healing comparisons use the same fractured configuration. Whiskers are retained as descriptive variability markers from the original post-processing and are not between-network standard deviations. Atom colors in panel (**a**) are carbon gray, oxygen red, and hydrogen white, with the simulation cell outlined in blue. In panel (**b**), 300 K is shown by black squares; 400 K without/with BER by red circles/blue upward triangles; and 500 K without/with BER by green downward triangles/purple diamonds. In panel (**c**), 300, 400, and 500 K are black, blue, and red, respectively; in panels (**d**,**e**), 400 and 500 K are blue and red. Faint traces in panel (**c**) show unsmoothed values around the darker trend lines, whereas panel (**d**) shows raw cumulative counts without an uncertainty band.

The self-healing behavior of the vitrimer was evaluated by analyzing the recovery of Young’s modulus (η) under different temperatures. The schematic diagram of the self-healing process is shown in Figure 7a. Healing simulations were carried out both with and without dynamic BERs to disentangle the respective roles of thermal motion and vitrimer chemistry. In the present nanoscale model, η quantifies recovery of the initial small-strain stiffness after fracture–surface reconsolidation; it does not imply full restoration of the original bulk network topology.

As shown in Figure 7b, *η* is strongly temperature-dependent. At 300 K, where the imposed BER probability is effectively zero and segmental mobility is limited, the apparent small-strain modulus recovers to approximately 31.6% after 0.1 ns, 69% after 2 ns, and 75.5% after 5 ns. At 400 K or above, the nonreactive systems approach approximately 98% recovery within 5 ns. This high nanoscale modulus recovery does not represent complete chemical healing: the damaged region is only a few nanometers wide, so re-establishment of local load transfer can restore small-strain stiffness without recreating the original bulk topology or macroscopic fracture resistance.

At 400 K, near the baseline overlap of T_g_ and T_BER_, nonreactive recovery is already enhanced by segmental interdiffusion, renewed van der Waals contacts, and possible re-entanglement. BER provides an additional early kinetic benefit: during 0 ns–2 ns, the average recovery with BER is approximately 8 percentage points higher than without BER. At longer times, the nonreactive system continues to recover and the two conditions converge, indicating that exchange mainly accelerates early recovery rather than increasing the final nanoscale modulus. At 500 K, reactive and nonreactive curves are nearly identical because thermal motion and interfacial reconsolidation dominate on the accessible timescale. The 400 K advantage is therefore conditional on the baseline BER schedule, not proof of a universal T_g_-centered optimum.

To further demonstrate that thermal motion is the primary driving force for the self-healing process, we analyzed the time evolution of van der Waals interaction energy and density in three nonreactive systems at different temperatures. Density convergence was employed to assess equilibration, which is especially critical for vitrimers because stress relaxation and network reorganization strongly influence mechanical stability and dynamic bond-exchange behavior [38]. As shown in Figure 7c, the trends of the van der Waals energy and density curves are highly consistent across all three temperatures. This agreement indicates that, in the absence of BER, the recovery process is governed by physical relaxation mechanisms, primarily interchain contact re-establishment, nanoscale interdiffusion, and densification driven by noncovalent interactions, rather than by chemical network rearrangement. In addition, a clear temperature dependence is observed in the relaxation times required for the systems to reach a stable state. At 300 K, the system does not fully equilibrate even after 5 ns, whereas stabilization occurs at approximately 3.8 ns at 400 K and is completed within only 0.2 ns at 500 K. This progressive reduction in relaxation time with increasing temperature correlates directly with the enhancement of η. These results demonstrate that elevated temperatures significantly accelerate chain mobility and interfacial reconsolidation, thereby promoting faster recovery of local mechanical stiffness across the fractured interface.

Additional mechanistic insight is obtained by analyzing the temporal evolution of reaction counts and reaction rates during healing. As shown in Figure 7d,e, the cumulative number of BERs increases continuously, while the reaction rate is initially high and then decreases to a lower quasi-steady level at all temperatures. However, comparison with the time evolution of η indicates that increasing reaction activity does not necessarily produce a proportional increase in the apparent modulus recovery at longer times. In particular, during the subsequent ~3 ns, η increases only gradually despite ongoing reactions, consistent with the preceding thermal-relaxation analysis and suggesting that physical relaxation and chain mobility remain rate-limiting in this regime.

Although the initial event rate at 500 K exceeds that at 400 K, the reactive system does not show a larger increment in apparent modulus recovery because the nonreactive 500 K interface already relaxes rapidly. In the baseline parameterization, BER therefore contributes most visibly when exchange attempts and segmental motion overlap while physical relaxation is not yet complete. Temperature-driven mobility dominates at higher temperature, and a different T_BER_ would shift the location of this overlap.

### 3.5. Model Scope and Limitations

Four scope conditions bound the interpretation. First, REACTER uses prescribed templates, geometric criteria, and acceptance probabilities; it does not calculate reaction free-energy barriers or absolute chemical rates. Second, the analytical p sensitivity establishes the expected 1/p scaling of the acceptance clock but does not demonstrate matched-conversion topology independence; all reported structures and properties remain conditioned on p = 1.0. Third, T_BER_ = 400 K is an imposed schedule parameter placed near the fitted T_g_ = 394 K, whereas the operational T_v_ is not calculated. Fourth, the controlled paired design uses one polymerized topology and one fractured geometry. This strengthens within-realization condition comparisons but does not quantify network-to-network variability. Together with the short atomistic timescale and nanoscale interface, these conditions mean that the reported trends are comparative and model-conditioned rather than predictions of macroscopic kinetics, population statistics, or universal transition temperatures.

## 4. Conclusions

We developed a chemically explicit, template-based all-atom molecular dynamics framework that follows Bis-GMA/2-HEMA polymerization and subsequent transesterification. REACTER permits direct monitoring of accepted topology-changing events and comparison of reactive and nonreactive responses, while the revised analysis clearly separates the calculated T_g_, the uncalculated operational T_v_, and the imposed BER reference temperature T_BER_.

For the production choice T_BER_ = 400 K, approximately 6 K above the fitted T_g_, BERs and emerging segmental mobility cooperate in the transition region, accelerating finite-time stress relaxation and early nanoscale modulus recovery. At 500 K, thermal chain mobility and physical interface reconsolidation dominate, and the incremental effect of BER is small. This crossover is therefore attributed to overlap between the prescribed exchange window and temperature-dependent mobility, not to an assumed physical identity of T_v_ and T_g_.

The simulations provide a molecular picture of how prescribed dynamic covalent exchange and physical relaxation can cooperate within an atomistic observation window. Exact endpoint accounting, analytical acceptance-probability sensitivity, and the controlled paired design define what can be inferred from the archived calculations without reconstructing unsupported trajectories. The results are most useful as comparative mechanistic guidance. Quantitative prediction of processing kinetics or network-population variability will require preserved event histories, validated reaction energetics, matched-conversion acceptance-probability calculations, and independently polymerized realizations.

## Data Availability

The original contributions presented in this study are included in the article/Supplementary Material. Further inquiries can be directed to the corresponding author.

## Supplementary Materials

The following supporting information can be downloaded at: https://www.mdpi.com/article/10.3390/ma19173702/s1, Figure S1: Bis-GMA and 2-HEMA monomers, representative polymerized and transesterified structures, and the corresponding atom-type labels used in the REACTER templates; Table S1: Force-field parameters for Bis-GMA and 2-HEMA; Table S2: System-level force-field checks and interpretation; Figure S2: Input-function sensitivity of the prescribed BER schedule. The production curve uses T_BER_ = 400 K, p_TBER_ = 0.90, and w = 20 K; the fitted Tg = 394 K is indicated by the vertical dashed line. The other curves shift T_BER_ by −40 K and +40 K while retaining the curve shape. These are analytical input translations, not new mechanical or healing simulations; Table S3: Expected qualitative consequences of shifting the imposed BER window; Figure S3: Analytical sensitivity of the REACTER acceptance gate. Lines show E[K] = pN, where N is the number of geometrically and topologically eligible trials. The horizontal line marks the connectivity-derived lower bound K = 653; the intersections give 653, 1306, and 2612 expected eligible trials for p = 1.00, 0.50, and 0.25. This is an analytical algorithmic sensitivity, not a reconstructed reaction-time trajectory; Table S4: Analytical acceptance-probability sensitivity for the lower-bound target K = 653 accepted inter-component events.
