# Supplementary material for: Molecular Insights into Dynamic Relaxation in Vitrimers: Transesterification-Driven Self-Healing at the Atomistic Level
Source: Materials (Basel). 2026 Aug 31;19(17):3702. doi: 10.3390/ma19173702 (PMC13567598; doi:10.3390/ma19173702)
Supplement: Supplementary file 1 [file materials-19-03702-s001.zip › materials-4507669-supplementary.pdf]

materials

MDPI

Supplementary Information

Molecular Insights into Dynamic Relaxation in Vitrimers: Transesterification-Driven Self-Healing at the Atomistic Level

Hao Yuan [1], Austin Knight [2], Long Jiang [2] and Liangliang Huang [1,*]

[1] School of Sustainable Chemical, Biological and Materials Engineering, University of Oklahoma, Norman, Oklahoma 73019, USA; hao.yuan-1@ou.edu
[2] Department of Mechanical Engineering, North Dakota State University, Fargo, North Dakota 58108, USA; austin.knight@ndsu.edu (A.K.); long.jiang@ndsu.edu (L.J.)
* Correspondence: hll@ou.edu

S1. Force-Field Atom Types and Reaction-Template Structures

To maintain the numerical stability of the Bis-GMA model while preserving overall electroneutrality, the hydroxyl hydrogen atom type HO1 was assigned zero charge and the corresponding OH1 oxygen charge was adjusted. In addition, polymerization and transesterification change local bonding and atom types through the pre- and post-reaction templates shown below. This charge modification is a model assumption and is included explicitly so that the force-field scope can be evaluated.

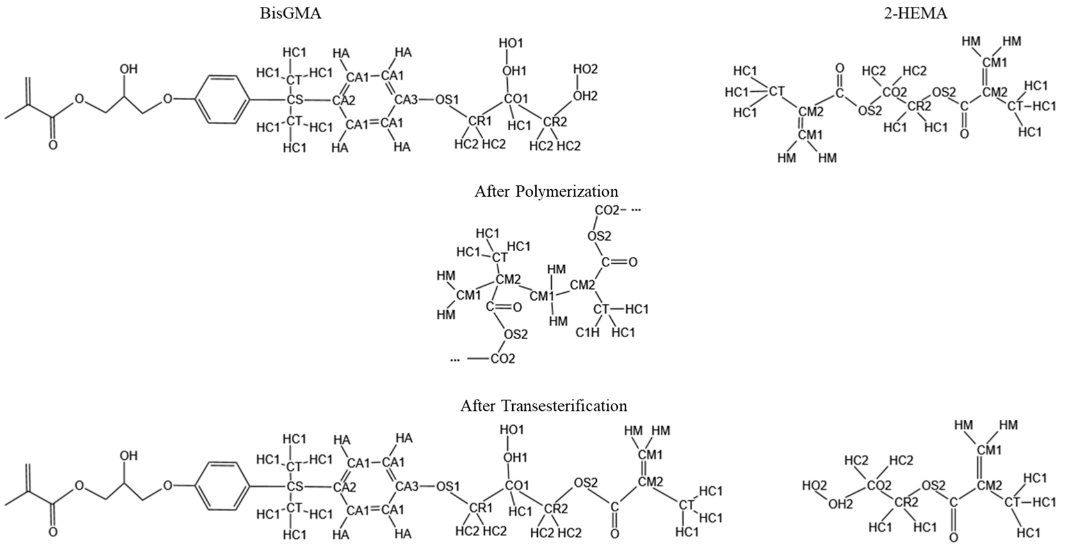

Figure S1. Bis-GMA and 2-HEMA monomers, representative polymerized and transesterified structures, and the corresponding atom-type labels used in the REACTER templates.

Table S1. Force-field parameters for Bis-GMA and 2-HEMA.

Atom Type	ε (kcal/mol)	σ (Å)	q×0.8
C	0.105	3.750	0.4080
CA1	0.070	3.550	-0.0920
CA2	0.070	3.550	0.0000
CA3	0.070	3.550	0.0680
CM1	0.076	3.550	-0.1840
CM2	0.076	3.550	0.0000
CO1	0.066	3.500	0.1640
CO2	0.066	3.500	0.1160
CR1	0.066	3.500	0.1120

CR2	0.066	3.500	0.1520
CS	0.066	3.500	0.0000
CT	0.066	3.500	-0.1440
HA	0.030	2.420	0.0920
HC1	0.030	2.500	0.0480
HC2	0.030	2.500	0.0240
HM	0.030	2.500	0.0920
HO1	0.000	0.000	0.0000*
HO2	0.000	0.000	0.3344
O	0.210	2.960	-0.3440
OH1	0.170	3.120	-0.2120
OH2	0.170	3.120	-0.5464
OS1	0.140	2.900	-0.2280
OS2	0.170	3.000	-0.2640

**Bond Type**	**K_r (kcal/mol Å^2)**	**r (Å)**	**Bond Type**	**K_r (kcal/mol Å^2)**	**r (Å)**
C-CM2	410	1.444	CO1-OH1	320	1.410
C-O	570	1.229	CO2-CR2	268	1.529
C-OS2	214	1.327	CO2-HC1	340	1.090
CA1-CA1	469	1.400	CO2-OH2	320	1.410
CA1-CA2	469	1.400	CR1-HC2	340	1.090
CA1-CA3	469	1.400	CR1-OS1	320	1.410
CA1-HA	367	1.080	CR2-HC2	340	1.090
CA2-CS	317	1.510	CR2-OS2	320	1.410
CA3-OS1	450	1.364	CS-CT	268	1.529
CM1-CM2*	268	1.529	CT-HC1	340	1.090
CM1-HM	340	1.090	HO1-OH1	553	0.945
CM2-CT	268	1.529	HO2-OH2	553	0.945
CO1-CR1	268	1.529	CO1-CO2	268	1.529
CO1-CR2	268	1.529	CR2-CR2	268	1.529
CO1-HC1	340	1.090			

**Angle Type**	**K_θ (kcal/mol rad^2)**	**θ (°)**	**Bond Type**	**K_θ (kcal/mol rad^2)**	**r (°)**
C-CM2-CM1	63.00	111.1	CR1-CO1-CR2	58.35	112.70
C-CM2-CT	63.00	111.1	CR1-CO1-HC1	37.50	110.7
C-OS2-CR2	83.00	116.9	CR1-CO1-OH1	50.00	109.5
CA1-CA1-CA2	63.00	120.0	CR2-CO1-HC1	37.50	110.7
CA1-CA1-CA3	63.00	120.0	CR2-CO1-OH1	50.00	109.5
CA1-CA1-HA	35.00	120.0	CR2-CO2-HC1	37.50	110.7
CA1-CA2-CA1	63.00	120.0	CR2-CO2-OH2	50.00	109.5
CA1-CA2-CS	70.00	120.0	CS-CT-HC1	37.50	110.7
CA1-CA3-CA1	63.00	120.0	CT-CS-CT	58.35	112.7
CA1-CA3-OS1	70.00	120.0	HM-CM1-HM	33.00	107.8
CA2-CA1-HA	35.00	120.0	HC1-CO1-OH1	35.00	109.5
CA2-CS-CA2	63.00	114.0	HC1-CO2-HC1	33.00	107.8
CA2-CS-CT	63.00	114.0	HC1-CO2-OH2	35.00	109.5
CA3-CA1-HA	35.00	120.0	HC1-CT-HC1	33.00	107.8
CA3-OS1-CR1	75.00	111.0	HC2-CR1-HC2	33.00	107.8
CM1-CM2-CT	58.35	112.0	HC2-CR1-OS1	35.00	109.5
CM2-C-O	80.00	120.4	HC2-CR2-HC2	33.00	107.8
CM2-C-OS2	81.00	111.4	HC2-CR2-OS2	35.00	109.5

CM2-CM1-HM	37.50	110.7	O-C-OS2	83.00	123.4
CM2-CT-HC1	35.00	109.5	CO1-CO2-OH2	50.00	109.5
CO1-CR1-HC2	37.50	110.7	CO1-CO2-HC1	37.50	110.7
CO1-CR1-OS1	50.00	109.5	CO2-CO1-CR1	58.35	112.7
CO1-CR2-HC2	37.50	110.7	CO2-CO1-HC1	37.50	110.7
CO1-CR2-OS2	50.00	109.5	CO2-CO1-OH1	50.00	109.5
CO1-OH1-HO1	55.00	108.5	CR2-CR2-HC2	37.50	110.7
CO2-CR2-HC2	37.50	110.7	CR2-CR2-OS2	50.00	109.5
CO2-CR2-OS2	50.00	109.5	CM1-CM2-CM1	58.35	112.7
CO2-OH2-HO2	55.00	108.50	CM2-CM1-CM2	58.35	112.7

**Dihedral Type**	**K1**	**K2**	**K3**	**K4**
C-CM2-CT-HC1	0.000	0.000	-0.186	0.000
CA1-CA1-CA2-CA1	0.000	7.250	0.000	0.000
CA1-CA1-CA2-CS	0.000	7.250	0.000	0.000
CA1-CA1-CA3-CA1	0.000	7.250	0.000	0.000
CA1-CA1-CA3-OS1	0.000	7.250	0.000	0.000
CA1-CA2-CS-CA2	0.000	7.250	0.000	0.000
CA1-CA2-CS-CT	0.000	0.000	0.000	0.000
CA1-CA3-OS1-CR1	0.000	3.000	0.000	0.000
CA2-CA1-CA1-CA3	0.000	7.250	0.000	0.000
CA2-CA1-CA1-HA	0.000	7.250	0.000	0.000
CA2-CS-CT-HC1	0.000	7.250	0.462	0.000
CA3-CA1-CA1-HA	0.000	7.250	0.000	0.000
CM1-CM2-CT-HC1	0.000	0.000	0.300	0.000
CM2-C-OS2-CR2	4.669	5.124	0.000	0.000
CO1-CR1-OS1-CA3	0.650	-0.250	0.670	0.000
CO1-CR2-OS2-C	-1.220	-0.126	0.422	0.000
CO2-CR2-OS2-C	-1.220	-0.126	0.422	0.000
CR1-CO1-CR2-HC2	0.000	0.000	0.300	0.000
CR1-CO1-CR2-OS2	1.711	-0.500	0.663	0.000
CR1-CO1-OH1-HO1	-0.356	-0.174	0.492	0.000
CR2-CO1-CR1-HC2	0.000	0.000	0.300	0.000
CR2-CO1-CR1-OS1	1.711	-0.500	0.663	0.000
CR2-CO1-OH1-HO1	-0.356	-0.174	0.492	0.000
CR2-CO2-OH2-HO2	-0.356	-0.174	0.492	0.000
CT-CS-CT-HC1	0.000	0.000	0.300	0.000
HA-CA1-CA1-HA	0.000	7.250	0.000	0.000
HA-CA1-CA2-CA1	0.000	7.250	0.000	0.000
HA-CA1-CA2-CS	0.000	7.250	0.000	0.000
HA-CA1-CA3-CA1	0.000	7.250	0.000	0.000
HA-CA1-CA3-OS1	0.000	7.250	0.000	0.000
HM-CM1-CM2-C	0.000	0.000	-0.225	0.000
HM-CM1-CM2-CT	0.000	0.000	0.300	0.000
HC1-CO1-CR1-HC2	0.000	0.000	0.300	0.000
HC1-CO1-CR1-OS1	0.000	0.000	0.468	0.000
HC1-CO1-CR2-HC2	0.000	0.000	0.300	0.000
HC1-CO1-CR2-OS2	0.000	0.000	0.468	0.000
HC1-CO1-OH1-HO1	0.000	0.000	0.352	0.000
HC1-CO2-CR2-HC2	0.000	0.000	0.300	0.000
HC1-CO2-CR2-OS2	0.000	0.000	0.468	0.000
HC1-CO2-OH2-HO2	0.000	0.000	0.352	0.000

HC2-CR1-OS1-CA3	0.000	0.000	0.760	0.000
HC2-CR2-OS2-C	0.000	0.000	0.198	0.000
O-C-CM2-CM1	0.000	0.000	0.000	0.000
O-C-CM2-CT	0.000	0.000	0.000	0.000
O-C-OS2-CR2	0.000	5.124	0.000	0.000
OH1-CO1-CR1-HC2	0.000	0.000	0.468	0.000
OH1-CO1-CR1-OS1	4.319	0.000	0.000	0.000
OH1-CO1-CR2-HC2	0.000	0.000	0.468	0.000
OH1-CO1-CR2-OS2	4.319	0.000	0.000	0.000
OH2-CO2-CR2-HC2	0.000	0.000	0.468	0.000
OH2-CO2-CR2-OS2	4.319	0.000	0.000	0.000
OS2-C-CM2-CM1	0.000	0.000	-0.553	0.000
OS2-C-CM2-CT	0.000	0.000	-0.553	0.000
CO1-CO2-OH2-HO2	-0.356	-0.174	0.492	0.000
CO2-CO1-OH1-HO1	-0.356	-0.174	0.492	0.000
HC1-CO1-CO2-OH2	0.000	0.000	0.468	0.000
OH1-CO1-CO2-OH2	9.508	0.000	0.000	0.000
CR1-CO1-CO2-OH2	-1.552	0.000	0.000	0.000
HC1-CO1-CO2-HC1	0.000	0.000	0.300	0.000
OH1-CO1-CO2-HC1	0.000	0.000	0.468	0.000
CR1-CO1-CO2-HC1	0.000	0.000	0.300	0.000
CO2-CO1-CR1-OS1	1.711	-0.500	0.663	0.000
CO2-CO1-CR1-HC2	0.000	0.000	0.300	0.000
HC2-CR2-CR2-HC2	0.000	0.000	0.300	0.000
HC2-CR2-CR2-OS2	0.000	0.000	0.468	0.000
CR2-CR2-OS2-C	-1.220	-0.126	0.422	0.000
OS2-CR2-CR2-OS2	-0.550	0.000	0.000	0.000
CM2-CM1-CM2-C	-1.697	-0.456	0.585	0.000
CM2-CM1-CM2-CM1	1.300	-0.050	0.200	0.000
CM2-CM1-CM2-CT	1.300	-0.050	0.200	0.000
HM-CM1-CM2-CM1	0.000	0.000	0.300	0.000

S2. Force-Field Validation and Scope

The OPLS-AA model was assessed with system-level observables that are available from the submitted simulations and literature. The uncured 1:2 molar Bis-GMA/2-HEMA mixture density was 1.08 g cm^{-3} at 300 K and 1 atm. Using molecular weights of 512.59 and 130.14 g mol^{-1} and literature pure-component densities of 1.16 and 1.07 g cm^{-3}, respectively, an ideal-volume estimate is 1.13 g cm^{-3}. The simulated value is therefore approximately 4.3% lower. The simulated glass-transition temperature of 394 K falls within the reported 383–433 K range for related vinyl-ester thermosets. These comparisons support semi-quantitative packing and thermal trends but do not validate absolute reaction kinetics, transition-state energetics, or every local hydrogen-bond interaction. The 0.8 charge scaling and the custom hydroxyl-charge modification listed in Table S1 remain model assumptions.

Table S2. System-level force-field checks and interpretation.

Observable	Simulation	Reference / estimate	Difference
Uncured 1:2 molar mixture density at 300 K, 1 atm	1.08 g cm^{-3}	1.13 g cm^{-3} ideal-volume estimate from Bis-GMA = 1.16 and HEMA = 1.07 g cm^{-3}	−4.3%
Polymerized density	1.17 g cm^{-3}	No composition-matched experimental value identified in the submitted sources	—
Glass-transition temperature	394 K	383 K–433 K for related vinyl-ester thermosets	Within reported range

Reference values: Pratap et al., Jpn. Dent. Sci. Rev. 2019, 55, 126–138; Sadler et al., J. Mater. Chem. A 2013, 1, 12579–12586.

S3. Sensitivity of the Imposed BER Activation Temperature

The production model uses a prescribed temperature-dependent acceptance function for BER with T_{BER} = 400 K, p_{TBER} = 0.90, and w = 20 K. The independently fitted glass-transition temperature is T_g = 394 K, so the production T_{BER} lies 6 K above T_g and places the acceptance window within the same broad transition region without asserting T_v = T_g. Figure S2 illustrates the direct input-function consequence of shifting T_{BER} to 360 K and 440 K while retaining p_{TBER} and w. Note that the curves are analytical translations of the prescribed probability function and are not additional molecular-dynamics trajectories.

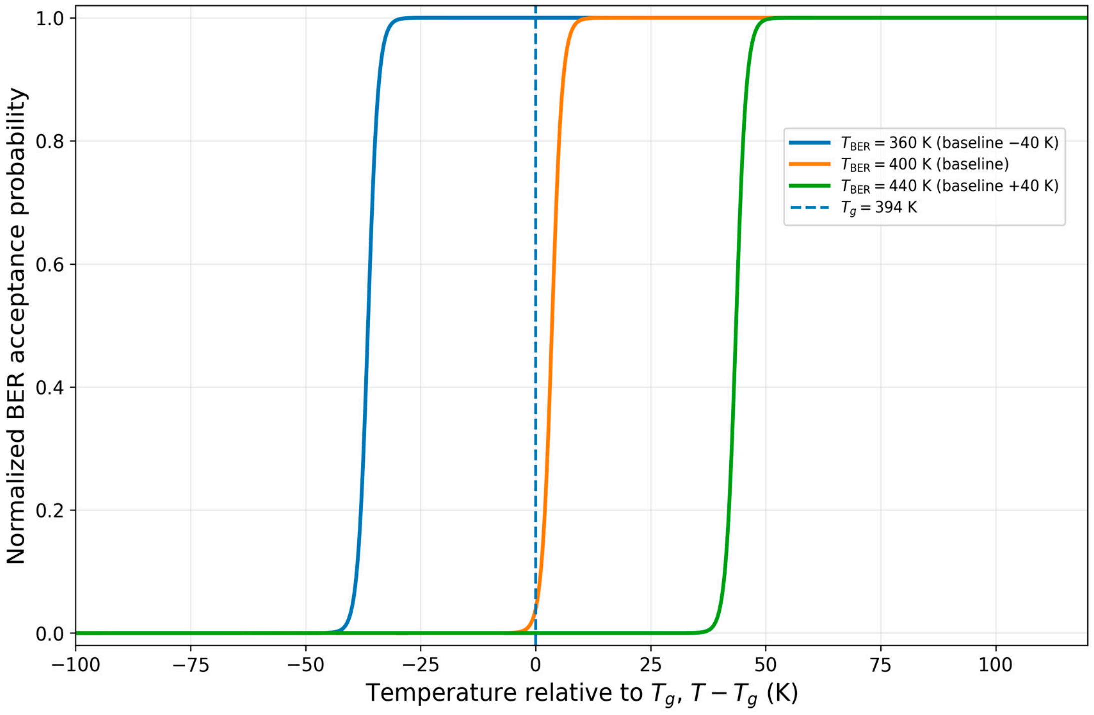

Figure S2. Input-function sensitivity of the prescribed BER schedule. The production curve uses T_{BER} = 400 K, p_{TBER} = 0.90, and w = 20 K; the fitted Tg = 394 K is indicated by the vertical dashed line. The other curves shift T_{BER} by −40 K and +40 K while retaining the curve shape. These are analytical input translations, not new mechanical or healing simulations.

Table S3. Expected qualitative consequences of shifting the imposed BER window.

Input schedule	BER acceptance relative to segmental mobility	Expected incremental BER effect	Interpretation
T_{BER} = 360 K (34 K below T_g)	Acceptance turns on in a more glassy regime	Early attempts may remain encounter- and mobility-limited; a visible property change can still emerge only as T approaches T_g	A lower activation input does not remove the segmental-mobility requirement.
T_{BER} = 400 K (production; 6 K above T_g)	Acceptance and emerging mobility overlap in the simulated transition region	The incremental BER contribution can be largest before high-temperature physical relaxation dominates	This is the schedule used for the reported production trajectories.
T_{BER} = 440 K (46 K above T_g)	Segmental mobility develops before substantial BER acceptance	Near-T_g reactive and nonreactive responses become more similar; BER-sensitive behavior shifts upward	The temperature of maximum BER contribution is model-dependent.

S4. Polymerization Endpoint Accounting and Acceptance-Probability Sensitivity

The additional analyses in this section use only quantities already reported in the manuscript and exact consequences of the REACTER acceptance rule. They are not interpolated molecular-dynamics trajectories. The calculation begins with 900 monomer components and ends after 5 ns with 247 connected components. Therefore, at least 653 intercomponent coupling events must have occurred. Intracomponent cyclization can increase the total accepted-event count without reducing component count, so 653 is a rigorous lower bound. The endpoint also contains approximately 891 participating monomers (99%), a density increase from 1.08 g cm^{-3} to 1.17 g cm^{-3}, and 23 components larger than 300 atoms. Because the intermediate fix bond/react history is not present in the archived files, no time-resolved polymerization curve is reconstructed.

For the REACTER acceptance gate, an eligible event is accepted with probability p. Under the ideal Bernoulli-trial representation, E[K] = pN for N eligible trials, and the expected number of eligible trials required to obtain K accepted events is E[N] = K/p. For the endpoint lower bound K = 653, reducing p from 1.00 to 0.50 or 0.25 increases the expected eligible-trial count by factors of two and four. The corresponding waiting-trial standard deviation is sqrt[K(1 − p)]/p. This analysis quantifies the construction-clock sensitivity of p; it does not demonstrate matched-conversion topology independence because accepted events alter subsequent geometry and eligibility.

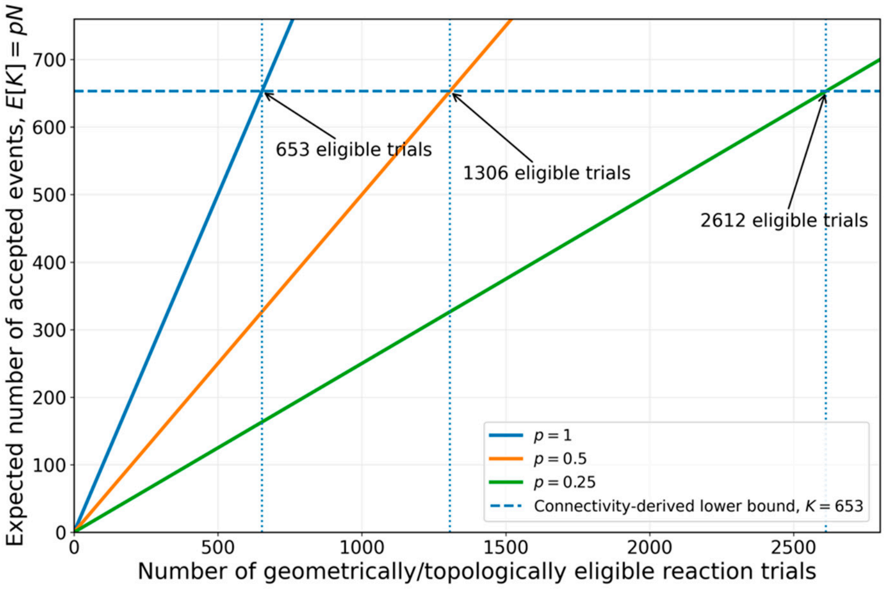

Figure S3. Analytical sensitivity of the REACTER acceptance gate. Lines show E[K] = pN, where N is the number of geometrically and topologically eligible trials. The horizontal line marks the connectivity-derived lower bound K = 653; the intersections give 653, 1306, and 2612 expected eligible trials for p = 1.00, 0.50, and 0.25. This is an analytical algorithmic sensitivity, not a reconstructed reaction-time trajectory.

Table S4. Analytical acceptance-probability sensitivity for the lower-bound target K = 653 accepted inter-component events.

p	Expected trials per accepted event (1/p)	Expected eligible trials for K = 653	SD of waiting trials	Interpretation
1.00	1.0	653	0	All eligible events are accepted; fastest construction clock.
0.50	2.0	1306	36.1	Expected trial count doubles; event ordering can still change as geometry evolves.
0.25	4.0	2612	88.5	Expected trial count quadruples; no matched-conversion topology equivalence is implied.

Note: The waiting-trial statistics follow a negative-binomial idealization with a fixed acceptance probability. In the actual reactive trajectory, the eligible-event population evolves after each accepted event; therefore, the table is an algorithmic first-order sensitivity rather than a substitute for matched-conversion simulations.
